# Accumulation of Metal-Specific T Cells in Inflamed Skin in a Novel Murine Model of Chromium-Induced Allergic Contact Dermatitis

**DOI:** 10.1371/journal.pone.0085983

**Published:** 2014-01-20

**Authors:** Hiroaki Shigematsu, Kenichi Kumagai, Hiroshi Kobayashi, Takanori Eguchi, Kazutaka Kitaura, Satsuki Suzuki, Tatsuya Horikawa, Takaji Matsutani, Kouetsu Ogasawara, Yoshiki Hamada, Ryuji Suzuki

**Affiliations:** 1 Department of Rheumatology and Clinical Immunology, Clinical Research Center for Rheumatology and Allergy, Sagamihara National Hospital, National Hospital Organization, Sagamihara, Japan; 2 Department of Oral and Maxillofacial Surgery, School of Dental Medicine, Tsurumi University, Yokohama, Japan; 3 Department of Oral and Maxillofacial Surgery, Nagano Matsushiro General Hospital, Nagano, Japan; 4 Department of Oral and Maxillofacial Surgery, Toshiba Rinkan Hospital, Sagamihara, Japan; 5 Section of Biological Science, Research Center for Odontology, Nippon Dental University, Tokyo, Japan; 6 Department of Dermatology, Nishi-Kobe Medical Center, Kobe, Japan; 7 Department of Immunobiology, Institute of Development, Aging and Cancer, Tohoku University, Sendai, Japan; Institute for Virus Research, Laboratory of Infection and Prevention, Japan

## Abstract

Chromium (Cr) causes delayed-type hypersensitivity reactions possibly mediated by accumulating T cells into allergic inflamed skin, which are called irritants or allergic contact dermatitis. However, accumulating T cells during development of metal allergy are poorly characterized because a suitable animal model is not available. This study aimed to elucidate the skewing of T-cell receptor (TCR) repertoire and cytokine profiles in accumulated T cells in inflamed skin during elucidation of Cr allergy. A novel model of Cr allergy was induced by two sensitizations of Cr plus lipopolysaccharide solution into mouse groin followed by single Cr challenge into the footpad. TCR repertoires and nucleotide sequences of complementary determining region 3 were assessed in accumulated T cells from inflamed skin. Cytokine expression profiles and T-cell phenotypes were determined by qPCR. CD3+CD4+ T cells accumulated in allergic footpads and produced increased T helper 1 (Th1) type cytokines, Fas, and Fas ligand in the footpads after challenge, suggesting CD4+ Th1 cells locally expanded in response to Cr. Accumulated T cells included natural killer (NK) T cells and Cr-specific T cells with VA11-1/VB14-1 usage, suggesting metal-specific T cells driven by invariant NKT cells might contribute to the pathogenesis of Cr allergy.

## Introduction

Metal allergy is categorized as a delayed-type hypersensitivity (DTH) reaction, and may be caused by metal ions released from dental materials, jewelry, and coins [1]. Recently, the number of patients with metal allergy has increased because metal is increasingly used for jewelry, surgical instruments, and dental restorations [1]. In addition to nickel (Ni), cobalt (Co), and palladium (Pd), which often induce metal allergy, chromium (Cr) has also been reported as a causal metal of allergic contact dermatitis. Cr hypersensitivity is one of the major occupational metal skin diseases especially in cement workers [2], [3]. Cr hypersensitivity is common in the general population with a prevalence of approximately 0.5% [4], [5], whereas the prevalence in cement workers is approximately 4.5% [4], [6].

Skin exposure to Cr produces both irritant (ICD) and allergic contact dermatitis (ACD). However, changes in immune response during contact dermatitis have not been elucidated because of its coexistence with the DTH reaction [7]. The irritant reaction does not require previous sensitization, and both types of contact dermatitis, despite their induction by different mechanisms, have not been differentiated by macroscopic appearance [8].

Because metal ions function as haptens, T cell-mediated responses can occur in metal allergy. Indeed, metal ions induce the proliferation of human T cells *in vitro* and limited TCR were observed in human T cells isolated from metal allergy patients [9]–[11]. Using peripheral blood mononuclear cells (PBMCs) obtained from patients of metal allergy, it was shown that T cells are involved in the development of metal allergy [9], [12]. Furthermore, oligoclonal T cells contribute to Cr allergy onset in metallic implant patients [13]. The nature of T cells that infiltrate tissues during the elicitation phase of DTH are thought to be autoreactive [14], yet their antigen specificity has not been determined. How pathogenic T cells at the sites of allergic inflammation contribute to the development of metal allergy has not been explored because a suitable animal model has not been established. On the basis of previous reports [15]–[17], we generated a novel murine model of metal allergy, and have found the accumulation of the metal specific T cells in inflamed skin [16], [17].

In the present study, we demonstrated that the Cr-induced murine model of ICD and ACD developed typical pathology. In addition, we investigated whether the TCR repertoire was skewed, and whether the cytokine profiles of accumulated T cells were specific in inflamed skin during ACD.

## Materials and Methods

### Ethics Statement

This study was performed in a strict accordance with recommendations in the Guidelines for Care and Use of Laboratory Animals set by the Clinical Research Center for Rheumatology and Allergy, Sagamihara National Hospital, Japan. All animal experiments were performed according to the relevant ethical requirements and with approval from the committees for animal experiments at the Clinical Research Center for Rheumatology and Allergy, Sagamihara National Hospital, Japan. All surgery was performed under tribromoethanol anesthesia, and all efforts were made to minimize suffering.

### Animals

BALB/cAJcl mice (5-week-old females) were obtained from CLEA Japan (Tokyo, Japan). Mice were maintained in standard aluminum cages (with a lid made of stainless-steel wire). Food and water were available *ad libitum*.

### Reagents

CrCl_2_ (purity>95%) was purchased from Wako Pure Chemical Industries (Osaka, Japan). lipopolysaccharide (LPS) from *Escherichia coli* (O55:B5) prepared by phenol–water extraction was purchased from Sigma (St Louis, MO, USA). CrCl_2_ and LPS were dissolved in sterile saline.

### Sensitization, Elicitation and Measurement of Irritant and Allergic Footpad Swelling

#### Sensitization

A total of 125 µl of 10 mM CrCl_2_ with 10 µg/ml LPS in saline was injected twice (at an interval of 7 days) intradermally (i.d.) into the left and right groin of mice (250 µl each). Seven days after sensitization, mice were challenged for the first time.

#### Challenge for elicitation

Non-sensitized mice (ICD mice) or sensitized mice (ACD mice) were challenged for elicitation with 25 µl of 10 mM CrCl_2_ (without LPS) in saline into the left and right footpad by i.d. injection under anesthesia with tribromoethanol. BALB/cAJcl mice sensitized with Cr plus LPS and then challenged with saline were used as controls. Footpad swelling was measured at the indicated times using a Peacock Dial Thickness Gauge (Ozaki MFG Co. Ltd, Tokyo, Japan).

### Immunohistochemical (IHC) Analyses

Footpads were obtained from Cr-induced ICD and ACD mice for histology and IHC analyses. Tissue samples were fixed with 4% paraformaldehyde-lysine-periodate overnight at 4°C. After washing with phosphate buffered saline (PBS), fixed tissues were penetrated by soaking in 5% sucrose/PBS for 1 h, 15% sucrose in PBS for 3 h, and then 30% sucrose in PBS overnight at 4°C. Tissue samples were embedded in Tissue Mount (Chiba Medical, Saitama, Japan) and snap-frozen in a mixture of acetone and dry ice. Frozen sections were sliced into 6 µm cryosections and air-dried on poly-L-lysine-coated glass slides. For histological analyses, the cryosections were stained with hematoxylin and eosin (H&E). For IHC analyses, antigen retrieval was performed. The cryosections were then stained with anti-mouse F4/80 (Cl-A3-1, Abcam, Cambridge, UK), CD3 (SP7, Abcam), CD4 (H129.19, Pharmingen, San Diego, CA, USA), and CD8α (53-6.7, Pharmingen) monoclonal antibodies (mAbs). Non-specific binding of mAbs was blocked by incubation of sections with PBS containing 1∶20 dilution of normal goat serum or normal rabbit serum, 0.025% Triton X-100 (Wako Pure Chemicals) and 5% BSA (Sigma–Aldrich) for 30 min at room temperature (RT). The sections were incubated with primary mAbs for 1 h at RT. After washing three times with PBS for 5 min, intrinsic peroxidase was quenched by 3% H_2_O_2_ in methanol. After soaking the sections in distilled water, they were washed twice. Sections were then incubated with a secondary antibody (biotinylated goat anti-hamster IgG antibody or biotinylated rabbit anti-rat IgG antibody) for 1 h at RT. After washing three times, the sections were incubated with Vectastain ABC Reagent (Vector Laboratories, Burlingame, CA, USA) for 30 min at RT, followed by 3,3′-diaminobenzidine (DAB) staining (0.06% DAB and 0.03% H_2_O_2_ in 0.1 M Tris-HCl buffer pH 7.6, Wako Pure Chemicals). Finally, the tissue sections were stained with hematoxylin to visualize the cell nuclei.

### Isolation of Total RNA from Tissues

Fresh footpads and spleens were obtained from mice and immediately soaked in RNAlater RNA Stabilization Reagent (Qiagen, Hilden, Germany). Total RNAs from footpads and spleens were extracted using the RNeasy Lipid Tissue Mini Kit (Qiagen) according to the manufacturer’s instructions.

### Quantitative PCR (qPCR)

The expression levels of mRNA for immune response-related genes including T cell-related CD antigens, cytokines, and cytotoxic granules were measured by qPCR using the Bio-Rad CFX96 system (Bio-Rad, Hercules, CA, USA). Specific primers for GAPDH, CD3, CD4, CD8, interferon (IFN)-γ, tumor necrosis factor (TNF)-α, interleukin (IL)-4, IL-5, Perforin, Granzyme A, Granzyme B, Fas ligand (Fas L), CXCL10, and CXCR3 were described previously [18], [19]. The following additional primers were designed for our study: CD14 (forward:5′-CATTTGCATCCTCCTGGTTTCTGA-3′, reverse:5′-GAGTGAGTTTTCCCCTTCCGTGTG-3′), IL-1β (forward:5′-CCCAAGCAATACCCAAAGAA-3′, reverse:5′-GCTTGTGCTCTGCTTGTGAG-3′), and Fas (forward:5′-AGGACTGCAAAATGAATGGG-3′, reverse:5′-AGGGTGCAGTTTGTTTCCAC-3′). Histidine decarboxylase (HDC)-specific primers and TNF-R1-specific primers were purchased from Takara Bio (Otsu, Japan): HDC (forward:5′- TCCATTAAGCTGTGGTTTGTGATTC-3′, reverse:5′- CGCTTCTGACCAGAGATTCAAAGTA-3′) and TNF-R1 (forward:5′- CAACGGCACCGTGACAATC-3′, reverse:5′- GGAGGTAGGCACAACTTCATACACT-3′). Freshly isolated total RNA from the footpads of mice was converted to cDNA using PrimeScript™ RT Reagent Kit (Takara Bio) according to the manufacturer’s instructions. The PCR reaction consisted of 5 µl of SsoFast™ EvaGreen® Supermix (Bio-Rad), 3.5 µl of RNase/DNase-free water, 0.5 µl of 5 µM primer mix, and 1 µl of cDNA in a final volume of 10 µl. Cycling conditions were as follows: 30 s at 95°C followed by 45 rounds of 1 s at 95°C and 5 s at 60°C. At the end of each run, melting curve analyses was performed from 65°C to 95°C to confirm the homogeneity of PCR products. All assays were repeated three times and the mean values were used for gene expression levels. Five points of tenfold serial dilutions of each standard transcript were used to determine the absolute quantification, specification and amplification efficiency of each primer set. Standard transcripts were generated by *in vitro* transcription of the corresponding PCR product into a plasmid. The nucleotide sequences were confirmed by DNA sequencing using the CEQ8000 Genetic Analysis System (Beckman Coulter, Fullerton, CA, USA). Sequence quality and concentration were validated using the Agilent DNA 7500 Kit in an Agilent 2100 Bioanalyzer (Agilent, Santa Clara, CA, USA). GAPDH gene expression was used as an internal control. The expression levels of each target gene were normalized to GAPDH expression.

### TCR Repertoire Analysis

TCR repertoire analysis was performed using samples from Cr induced ACD mice (n = 5) and control mice (n = 5) by adaptor ligation-mediated PCR and microplate hybridization assay [20]–[22]. Briefly, total RNA was converted to double-stranded cDNA using a Superscript cDNA synthesis kit (Invitrogen, Carlsbad, CA) according to the manufacturer’s instructions, except that a specific primer (BSL-18E) was used [21]. The P10EA/P20EA adaptors were ligated to the 5′ end of the cDNA, and this adaptor-ligated cDNA was cut with *Sph*I. PCR was performed with TCRα-chain constant region-specific or TCRβ-chain constant region-specific primers (MCA [TCR α-chain C region-specific primer] 1 or MCB [TCR β-chain C region-specific primer] 1) and P20EA. The second PCR was performed with MCA2 or MCB2 and P20EA. The third PCR was performed using both P20EA and 5′-biotinylated MCA3 or MCB3 primers for the biotinylation of PCR products. Ten picomoles of amino-modified oligonucleotides specific for the TCRAV (TCR α-chain variable) and TCRBV (TCR β-chain variable) segments were immobilized onto carboxylate-modified 96-well microplates with water-soluble carbodiimide. Prehybridization and hybridization were performed in GMCF buffer (0.5 M Na_2_HPO_4_, [pH 7.0], 1 mM EDTA, 7% SDS, 1% BSA and 7.5% formamide) at 47°C. One-hundred microliters of the denatured 5′-biotinylated PCR products were mixed with an equivalent volume of 0.4 N NaOH/10 mM EDTA, and the mixture added to 10 ml of GMCF buffer. One-hundred microliters of hybridization solution was used in each well of the microplate containing immobilized oligonucleotide probes specific for V segments. After hybridization, the wells were washed four times with washing buffer (2×SSC, 0.1% SDS) at RT. The plate was incubated at 37°C for 10 min for stringency washing. After washing four times with the same washing buffer, 200 µl of TB-TBS buffer (10 mM Tris-HCl, 0.5 M NaCl, pH 7.4, 0.5% Tween-20 and 0.5% blocking reagent; Roche Diagnostics, Basel, Switzerland) was added to block non-specific binding. Next, 100 µl of 1∶2,000-diluted alkaline phosphatase-conjugated streptavidin in TB-TBS was added, and the sample was incubated at 37°C for 30 min. Plates were washed six times in T-TBS (10 mM Tris-HCl, 0.5 M NaCl, pH 7.4, 0.5% Tween-20). For color development, 100 µl of substrate solution (4 mg/ml *p*-nitrophenylphosphate [Sigma–Aldrich], in 10% diethanolamine, pH 9.8) was added, and absorbance at 405 nm determined. The ratio of the hybridization intensity of each TCRV (TCR V region)-specific probe to that of a TCRC-specific probe (V/C value) was determined using the TCR cDNA concentrated samples that contained the corresponding V segment and the universal C segment, respectively. Absorbance obtained with each TCRV-specific probe was divided by the corresponding V/C value. The relative frequency was calculated using the corrected absorbencies by the formula: relative frequency (%) = (corrected absorbance of TCRV-specific probe/the sum of corrected absorbencies of TCRV-specific probes)×100.

### Determination of CDR3 Nucleotide Sequences

PCR was performed with 1 µl of 1∶20 diluted second PCR product, using a forward primer specific for the variable region and a reverse primer specific for the constant region (MCA4 or MCB4) under the conditions described above. The primers used in this study were as follows: AV11-1∶5′-CTGGAGGACTCAGGCACTTACT-3′, AV14-1∶5′-CAGGCAAAGGTCTTGTGTCC-3′, BV8-2∶5′-GGCTACCCCCTCTCAGACAT-3′ BV14-1∶5′-TTCATCCTAAGCACGGAGAAG-3′. PCR products eluted from the agarose gel were cloned into the pGEM-T Easy Vector (Promega, Madison, WI, USA). The recombinant plasmid DNA was transfected into DH5α competent cells. Sequence reactions were performed with the Genome Lab DTCS Quick Start Kit (Beckman Coulter) and analyzed by the CEQ8000 Genetic Analysis System. A total of 617 clones from the footpads of Cr-induced allergic mice were examined (n = 5).

### Statistical Analysis

Differences were analyzed statistically using the Student’s unpaired *t*-test using StatView 5.0 for Windows (SAS Institute Inc., Cary, NC). A *P* value <0.05 was determined to be statistically significant.

## Results

### Footpad Swelling in Cr-induced ICD and ACD Mice

**Figure 1:**
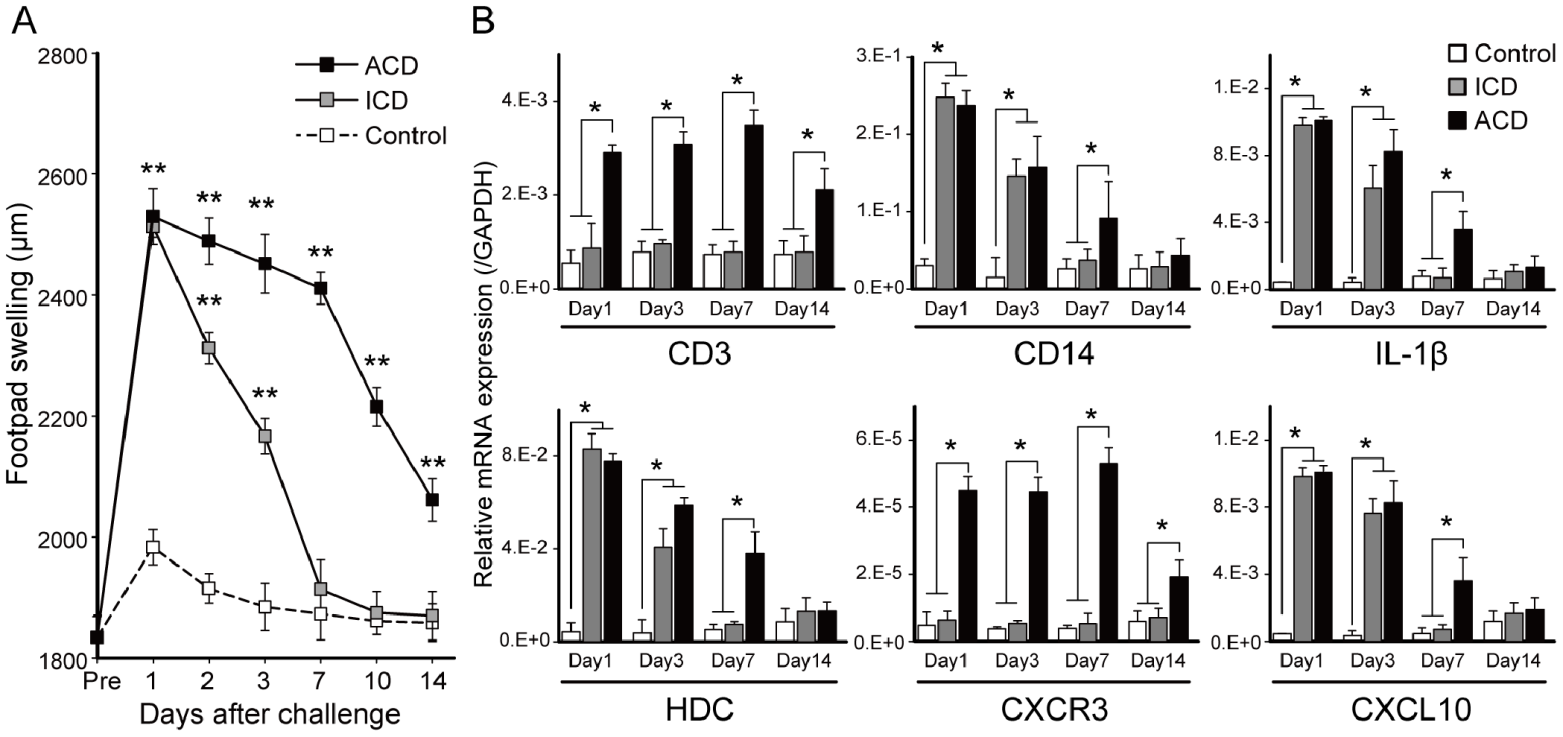
Footpad swelling and mRNA expression of inflammatory cell markers in Cr-induced ICD and ACD mice. (A) Footpad swelling at various time points. ICD, ACD, and control mice were analyzed at 1, 2, 3, 7, 10, and 14 days after challenge. (B) Footpad mRNA expression levels of IL-1β, HDC, CXCL10, CXCR3, CD14, and CD3 were assessed at the indicated days. Bars and error bars indicate mean ± SD. Statistical significance was tested by unpaired Student’s t-test (*p<0.05, **p<0.005).

To address how accumulated inflammatory cells in inflamed skin contributed to the development of metal ICD and ACD, we generated a novel murine model of ICD (n = 5) and ACD (n = 5) (Figure 1A). We used Cr plus LPS-sensitized and saline-challenged mice as controls. Footpad swelling of BALB/c mice sensitized with saline and then challenged with saline was similar with control mice (data not shown). Footpad swelling was measured every day after Cr injection into the footpad. The peak of footpad swelling was observed 1 day after the challenge in all mice. The footpad swelling at 1 day after challenge was similar in the ICD and ACD mice. Footpad swelling was reduced to basal levels 7 days after challenge in the ICD mice. In contrast, footpad swelling continued for 14 days after challenge in the ACD mice. We repeatedly performed the challenge, but footpad swelling was not enhanced (data not shown).

### Expression of Inflammatory Cell Markers in the Footpads of Cr-induced ICD and ACD Mice

To examine the accumulation of inflammatory cells into irritant and allergic inflamed skin, the mRNA expression levels of inflammatory cell markers were measured by qPCR. We compared IL-1β, HDC, CXCL10, CXCR3, CD14, and CD3 expression levels in ICD, ACD and control mice. In the ACD mice, CD3 and CXCR3 levels were significantly higher than the other mice at 1, 3, 7 days after challenge (Figure 1B). In contrast, expression levels of IL-1β, HDC, CXCL10, and CD14 in the ACD and ICD mice were significantly higher than the control mice (n = 5) at 1 and 3 days after challenge (Figure 1B).

### Histological and IHC Analyses of F4/80 and CD3 in Footpads of Cr-induced ICD and ACD Mice

**Figure 2:**
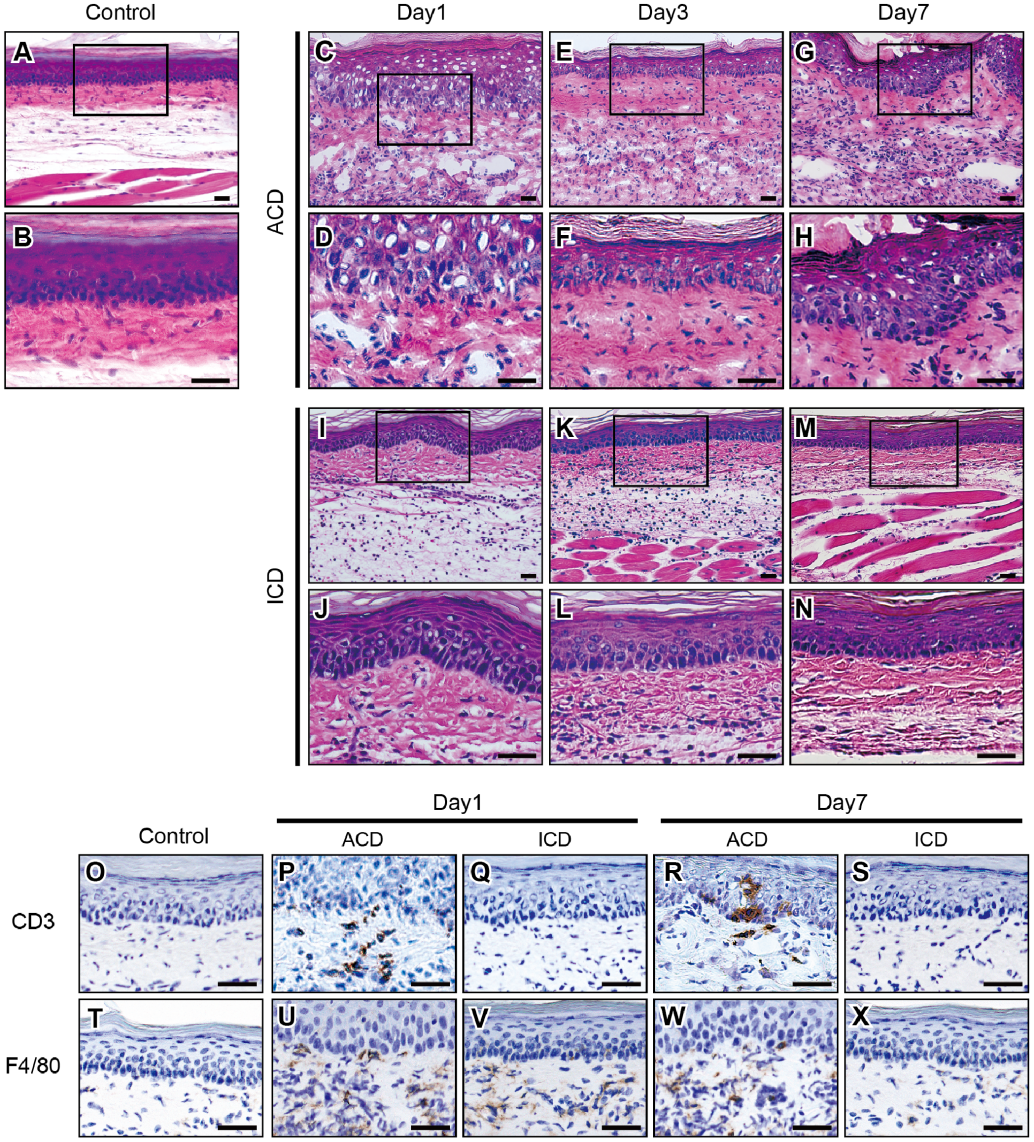
Histopathology and IHC analyses of CD3 and F4/80 in Cr-induced ICD and ACD mouse footpads. Histopathology and IHC analyses of F4/80+ and CD3+ cells in footpad tissues. Frozen sections of footpad tissues were prepared from ICD, ACD, and control mice at 1, 3, and 7 days after challenge. Sections were stained with H&E (A-N), CD3 (O-S), and F4/80 (T-X). Scale bar = 40 µm.

To verify whether macrophages and T cells infiltrated into inflamed skin, we analyzed the footpad skin of Cr-induced ICD and ACD mice and control mice at 1, 3, and 7 days after challenge (Figure 2). H&E staining showed dense mononuclear infiltrates in the epithelial basal layer and upper dermis, and liquefaction degeneration of the epithelial basal layer in the ICD and ACD mice at 1 day after challenge. In the ACD mice, inflammatory cells accumulated around the superficial venular bed and extended into the epidermis. Epidermal keratinocytes were partially separated, creating spongiotic dermatitis (Figure 2C, D). The inflammatory reaction in the footpads was diminished in the ICD mice (Figure 2I–N). In contrast, inflammation of the footpad remained in the ACD mice during the course of 7 days after challenge (Figure 2C–H).

Immunohistochemical (IHC) staining showed that CD3+ T cells were also present in the epithelial basal layer and the upper dermis, but not in the ICD or control mice (Figure 2O–S). F4/80+ macrophages were present in the epithelial layer in the ICD and ACD mice at 1 day after challenge (Figure 2U, V). At 7 days after challenge, F4/80+ macrophages were only present in the epithelial layer of the ACD mice (Figure 2W, X).

### Expression of T cell Markers and IHC Analyses in Footpads of Cr-induced ACD Mice

**Figure 3:**
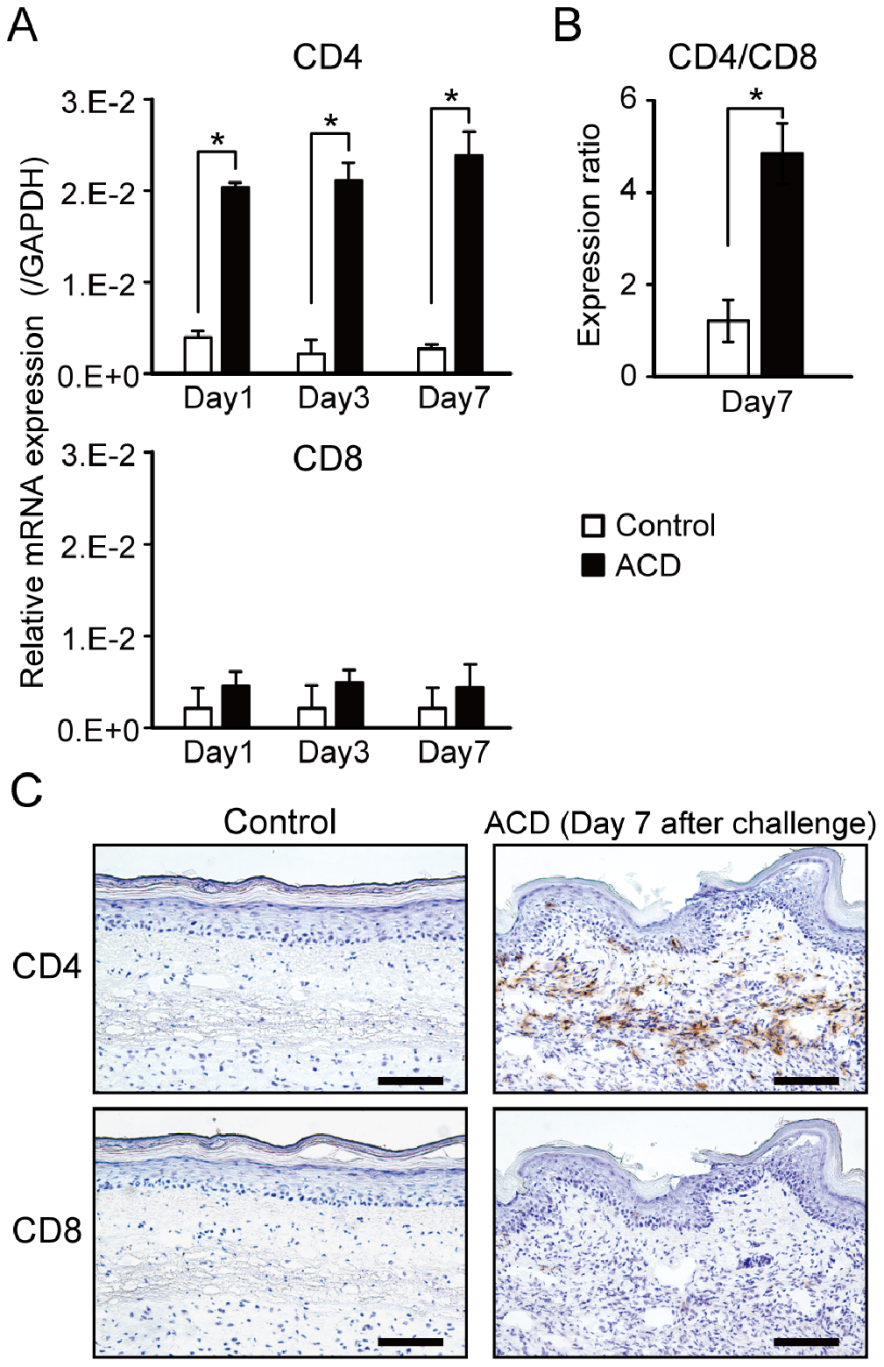
mRNA expression of T cell markers and IHC analyses in Cr-induced ACD mice. (A) Footpad mRNA expression levels of CD4 and CD8 at indicated days. (B) The expression ratio of CD4 to CD8 at 7 days after challenge. Bars and error bars indicate mean ±SD. Statistical significance was tested by unpaired Student’s t-test (*p<0.05) (C) IHC analyses (brown color) of CD4 and CD8 in footpads of Cr-induced ACD mice. Scale bar = 100 µm.

CD4 and CD8 expression were measured by qPCR and IHC as CD3+ T cells had infiltrated into inflamed skin in the ACD mice. In the ACD mice, CD4 levels were significantly higher than control mice (Figure 3A). However, CD8 levels were not significantly different between skin from ACD and control mice (Figure 3A). The expression ratio of CD4 to CD8 in the ACD mice was higher than control mice at 7 days after challenge (Figure 3B).

IHC staining showed that CD4+CD8− T cells were present in the epithelial basal layer and the upper dermis, at 7 days after challenge (Figure 3C). Infiltration of CD4+CD8− T cells into the inflamed skin was maximal at 7 days after challenge (data not shown).

### TCR Repertoires in Skin of Cr-induced ACD Mice

**Figure 4:**
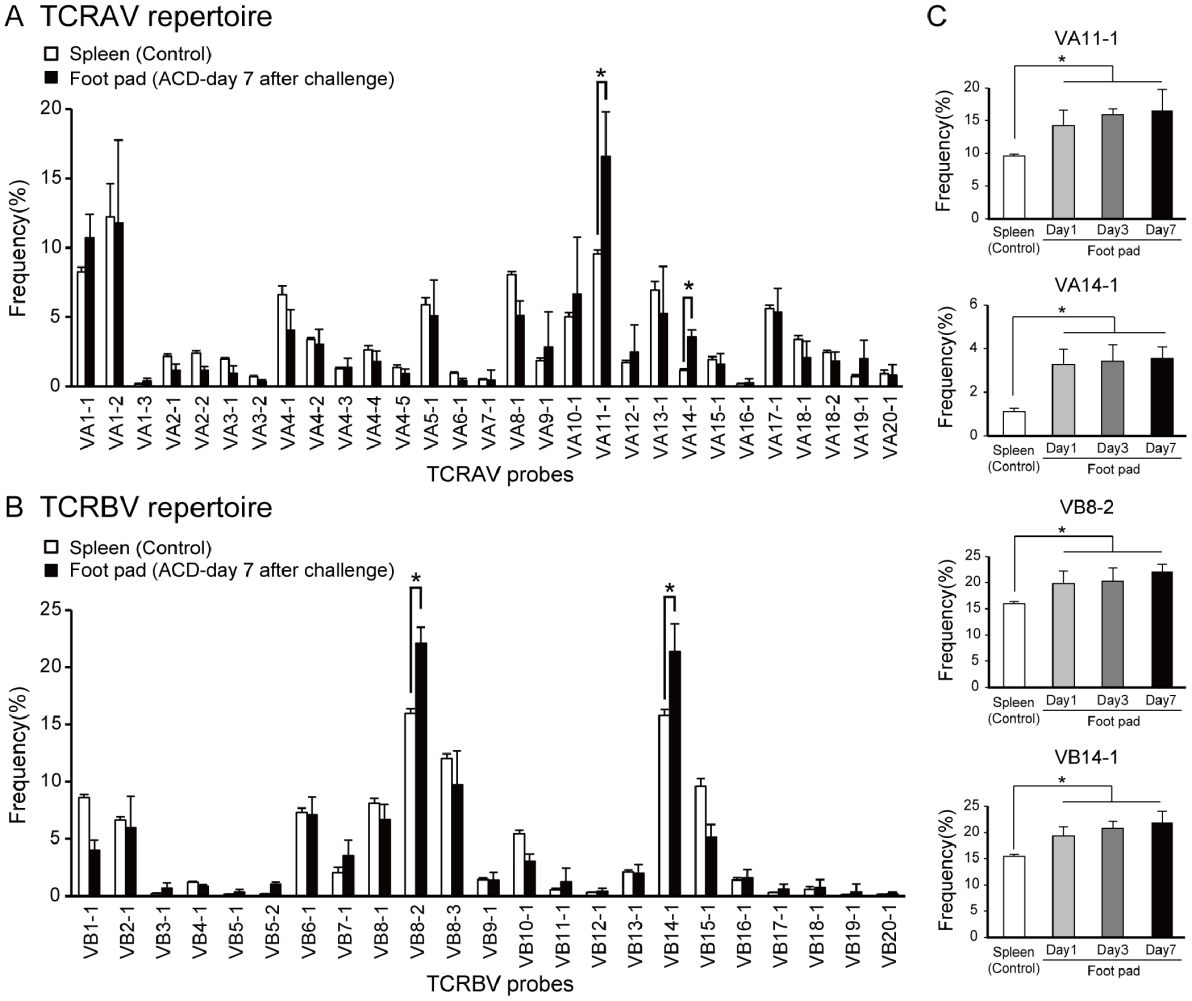
TCR repertoire analyses in Cr-induced ACD mice. (A, B) TCRAV and TCRBV repertoires were analyzed from footpads of ACD and control mice by microplate hybridization assay. Spleens from corresponding Cr-injected mice and saline-injected mice were used as controls. Bars and error bars indicate mean ±SD. At 7 days after challenge, percentage frequencies of the expression levels of VA11-1, VA14-1, VB8-2, and VB14-1 were significantly higher in footpads of the ACD mice compared with the control mice (*p<0.05, unpaired Student *t*-test). (C) Increase in the frequencies of VA11-1, VA14-1, VB8-2, and VB14-1 after Cr challenge. TCRAV and TCRBV repertoires were analyzed from RNA samples from footpads of Cr-induced ACD mice obtained at day 1, 3 and 7. The frequencies of VA11-1, VA14-1, VB8-2, and VB 14-1 in the Cr-induced ACD mice were significantly increased at days 1, 3, and 7 compared with control mice (*p<0.05, unpaired Student *t*-test). All experiments were performed in triplicate.

To examine whether TCR repertoires were skewed in inflamed skin, we analyzed TCRAV and TCRBV repertoires in Cr-induced ACD mice (Figure 4). The percentage usage of VA11-1, VA14-1, VB8-2, and VB14-1 was significantly higher in the footpads of Cr-induced ACD mice compared with spleens from the control mice (Figure 4A, B). There were no significant differences in the percentage usage of other TRAVs and TRBVs in Cr-induced mouse footpads and control mice (Figure 4A, B). The expression of VA11-1, VA14-1, VB8-2, and VB14-1 increased gradually in the footpads over 1 week (Figure 4C), indicating that the increased expression correlated with the accumulation of CD4+ T cells.

### Determination of CDR3 Nucleotide Sequences in Cr-induced ACD Mice

**Figure 5:**
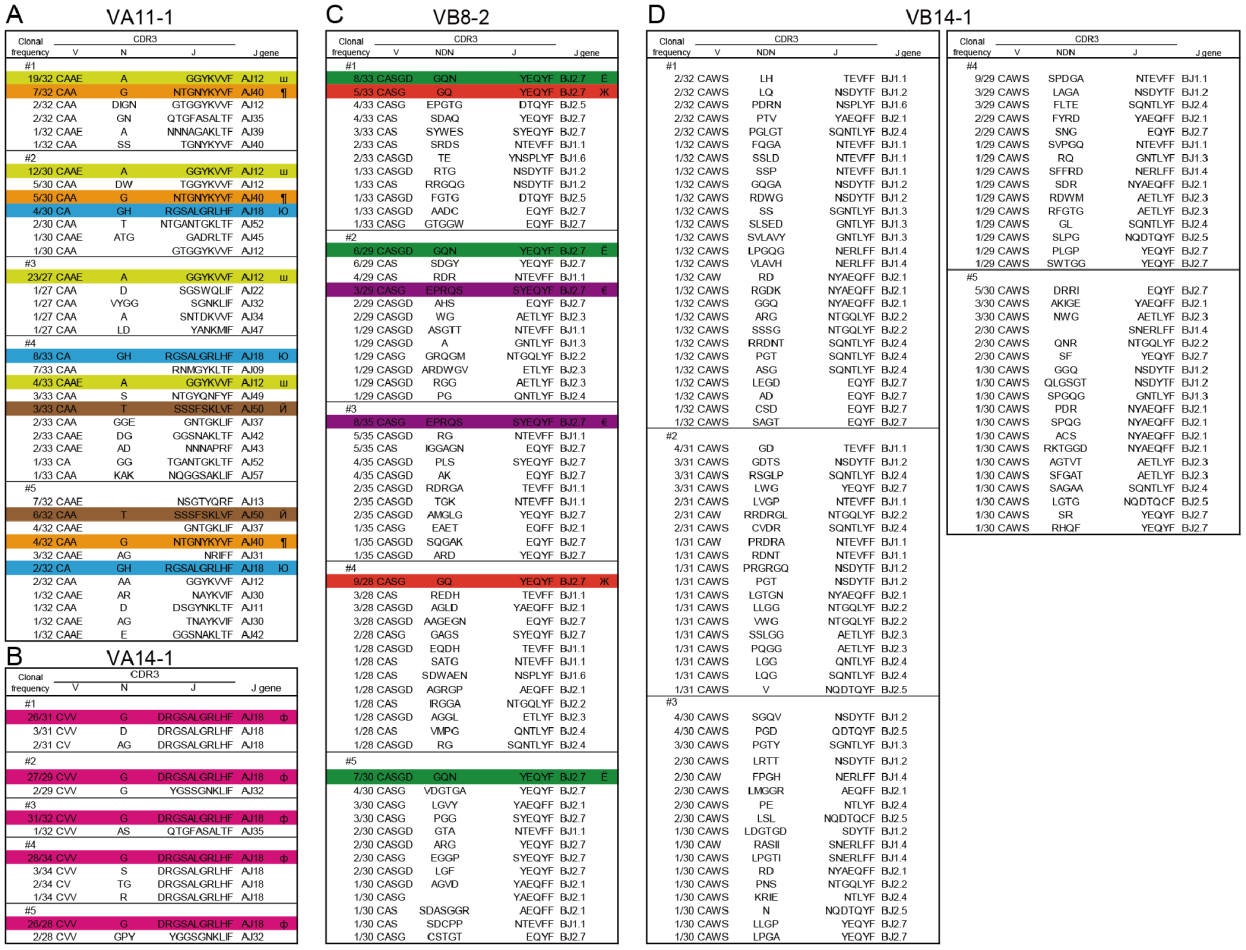
Characteristics of CDR3 regions of AV11-1, AV14-1, BV8-2 and BV14-1 in Cr-induced ACD mice. Amino acid sequences of CDR3 regions of VA11-1 (A), VA14-1 (B), VB8-2 (C), and VB14-1 (D) in Cr-induced ACD mouse footpads at 7 days after challenge. Identical CDR3 sequences (III, ¶, Ю, Й, ф, Ё, Ж, €) were obtained from the footpads of different mice (A–C).

To investigate whether specific CDR3 sequences were used in accumulated T cells bearing VA11-1, VA14-1, VB8-2, and VB14-1 we determined the CDR3 nucleotide sequences from cDNA clones obtained from footpads. Sequence analyses showed that identical TCR clonotypes bearing VA11-1, VA14-1, and VB8-2 were obtained from the footpads of different mice while diverse TCR clonotypes with VB14-1 were obtained from the footpads of all mice (Figure 5A–D). Surprisingly, CDR3 sequences of VA14-1 cDNA clones was most frequently utilized in the accumulated NKT cells and a clonotype (CVV-G-DRGSALGRLHFG) of CDR3 was obtained from the footpads in all Cr-induced ACD mice (Figure 5B). This clonotype had identical CDR3 sequences and common AJ18 segments (Figure 5B). In addition, we did not observe any motif for Cr-binding in CDR3α of VA11-1 but shared TCR clonotypes contained Glu (E), Thr (T), His (H), Arg (R), and Tyr (Y) residues (Table S1).

### Expression Levels of T cell Related Cytokines, Cytotoxic Granules, and Apoptosis Related Genes in Footpads of Cr-induced ACD Mice

**Figure 6:**
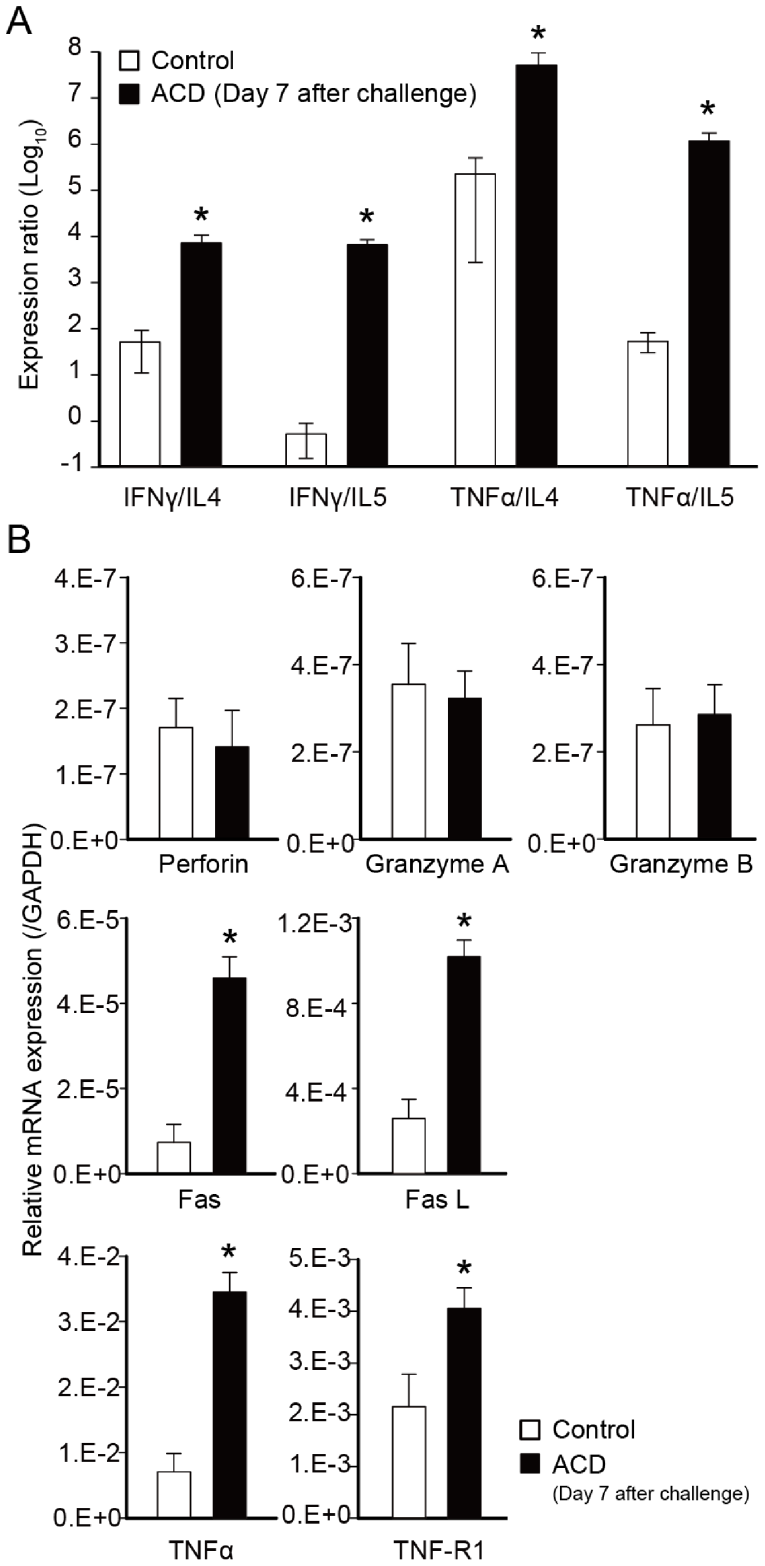
Expression levels of T cell cytokines, cytotoxic granules, and apoptosis-related genes in Cr-induced ACD mice. (A) The expression ratio of Th1-related/Th2-related genes. (B) The mRNA expression of cytotoxic granules (Perforin, granzyme A, and granzyme B), apoptosis related genes (Fas and Fas L), and TNF related genes (TNF-α, and TNF-R1) (*p<0.05, unpaired Student *t*-test). Bars and error bars indicate mean ±SD.

We compared ratios of the expression levels of Th1-related genes to those of Th2-related genes (IFN-γ/IL-4, IFN-γ/IL-5, TNF-α/IL-4, and TNF-α/IL-5) in footpads of Cr-induced ACD mice (Figure 6A). In the ACD mice, expression levels of Th1/Th2 ratios were significantly higher than in the control mice (Figure 6A). The expression levels of apoptosis-related genes (Fas and Fas L), TNF-α, and TNF-R1 were significantly increased in Cr-induced ACD mice than in control mice (Figure 6B). In contrast, cytotoxic granule (Perforin, Granzyme A and Granzyme B) expression was not significantly different in the skin of ACD and control group mice (Figure 6B).

## Discussion

This study demonstrated the successful establishment of a murine model of Cr-induced ICD and ACD with appropriate pathology. In addition, we observed that allergen specific T cells used a specific TCR repertoire in Cr-induced ACD mice. In previous reports, skin exposure with Cr caused both ICD and ACD [23]-[25], and it was suggested that keratinocytes were the first target cells affected by Cr [7]. ICD is a non-specific inflammatory dermatitis, mainly caused by the toxicity of chemicals on skin cells, which triggers inflammation by activation of the innate immune system [26]. ACD corresponds to a delayed-type hypersensitivity response, and skin inflammation is mediated by antigen-specific T cells [26]. We suggest that both types of contact dermatitis could not be differentiated by macroscopic appearance, as footpad swelling was the same in both mice at 1 day after challenge.

The mRNA expression of inflammatory cell markers in the footpads of Cr-induced ICD and ACD mice were different by qPCR analyses. In ACD mice, the expressions of T cell related genes (CD3 and CXCR3) were significantly higher than in other mice. This suggested the existence of T cells was the major cause of pathogenesis in ACD. In contrast, the expression of IL-1β, HDC, CXCL10, and CD14 was up regulated in both ACD and ICD. Thus, both mice developed acute edema induced by inflammatory cells at 1 day after challenge. Previous *in vivo* and *in vitro* studies demonstrated that haptens activate skin cells and induce the production of several cytokines including IL-1β that stimulates the migration of skin dendritic cells to the lymph nodes and has a key role in contact dermatitis [27]. Hapten-induced effects mediated by innate immune systems are essential for the activation of cutaneous antigen-presenting cells [28]. Indeed, the innate immunity response is a prerequisite for the activation of the ACD immune response [29].

LPS stimulates innate immunity via Toll-like receptor 4 (TLR4), and TLRs may modify adaptive immunity [30]. Recently, Schmidt et al. reported that Ni directly stimulates human TLR4, but not mouse TLR4, which may be crucial for the development of contact allergy [31]. In this study, we identified the accumulation of T cells in the footpads of Cr plus LPS sensitized–Cr-challenged mice compared with those of Cr without LPS sensitized–Cr-challenged mice. These results suggest that LPS also plays an important role of adjuvant to induce the metal specific T cells in the inflamed skin of Cr-induced ACD mice.

T cells bearing CXCR3 belong mainly to the CD4+ Th1 cell subset, and participate in the pathogenesis of ACD [32]. From 24 to 72 h after challenge, the infiltrates showed increased CD4/CD8 ratios of 2∶1 with 25–50% of infiltrating cells expressing CXCR3 [33]. Our results suggested the expression of CXCR3 ligands (such as CXCL10) by epidermal and dermal cells contributed to an environment in which activated T cells bearing CXCR3 migrated to the footpads of ACD mice.

Histological analysis showed apparent spongiosis in the inflamed footpad skins of ACD mice. Spongiosis is a characteristic histopathological feature in acute eczema [34]. In the initial 24 h following re-exposure to the hapten, numerous lymphocytes and macrophages accumulate to the superficial venular bed and extend into the epidermis. The epidermal keratinocytes are partially separated, creating spongiotic dermatitis. F4/80 is an monoclonal antibody target on the surface of mature macrophages and epidermal Langerhans cells [35], [36]. F4/80+ cells were stained in the epithelial basal layer of ACD and ICD mice at 1 day after challenge suggesting that inflammation in the footpads was initiated by the response of macrophages to Cr. IHC staining showed that CD4+ T cells were only present among the infiltrating cells in the epithelial basal layer in the ACD mice, but not the ICD mice. Inflammatory reactions in the foot pad were relatively diminished in ICD mice compared with ACD mice after challenge. This suggested the subsequent requirement of T cells for sustained inflammation, resulting in the delayed resolution of inflammatory reactions in ACD mice. Thus, T cells are recruited to the allergic sites in ACD lesions. Metal allergy can be elicited by either CD4+ or CD8+ T cells depending on the pathway by which antigen is processed [37], [38]. CD4+ T cells accumulated in large numbers in ACD, but not in ICD in the Cr-induced mouse model. These results suggest that sensitization occurred and Cr-specific CD4+T cells were induced in the ACD mice. Furthermore, the infiltrating T cells reacted to the self-antigens presented by major histocompatibility class (MHC) II antigens.

The infiltrating T cells from Cr-induced ACD mice expressed CD4+ and used a specific TCR repertoire expressing TCR VA11-1, VA14-1, VB8-2, and VB14-1. This study provides evidence for the first time that specific TCR-expressing T cells expand in response to Cr in the inflamed skin of mice. In addition, TCR VA14Jα18 and VB8-2 were a major subset of mouse NKT cells (also called invariant (i)NKT cells or Vα14i NKT cells) [39]. NKT cells are thought to recognize endogenous glycolipids and secrete cytokines (such as IFN-γ) that help to amplify early immune responses [40]. In addition, we have identified the accumulation of NKT cells in the lesional skin of Ni allergy [16], [41]. These results imply that NKT cells participate in cross-reactivity of metal allergy. As earlier reported in ACD, NKT cells were recruited with T cells into the inflamed skin [42]. Interestingly, we observed that Cr-specific T cells bearing TCR VA11-1 and VB14-1 accumulated in the inflamed skin of Cr-allergic mice. CDR3 sequence analysis revealed that most TCR clonotypes observed in Cr-induced allergy mice had a preferential usage of AJ segments (AJ12, 18, 40, 50 for VA11-1). In contrast to the very restricted TRAV repertoire, footpad-infiltrating T cells exhibited a relatively broad TRBV repertoire. The VB14-1, CDR3 sequences varied considerably among individual mice. This contrasting result provides an important insight into understanding antigen recognition by Cr-specific T cells.

Metal ions can act as haptens that covalently bind to proteins or peptides. Metal ions form geometrically highly defined, coordinated complexes with self-proteins, creating metal ion-protein complexes that can be processed by antigen-presenting cells and presented by MHC molecules [43], [44]. Thus, Cr-specific TCRs can recognize a hapten-modified peptide presented by MHC and therefore, similar to conventional peptide antigens, both CDR3α and 3β may be essential for antigen recognition. However, we observed diverse CDR3β sequences in VB14-1.

The role and significance of recognition of metal ions by TCRs might be different between CDR3α and CDR3β. CDR3β of Vβ5-1+ TCR had a dominant role in recognition of Beryllium (Be), with little contribution from TCRα chains [45]. This difference was explained by the TCR footprint being dominated by the TCRβ chain and β1 α-helix residues of HLA-DP2. Yin et al also reported CDR3β had a major role in recognition of human Nickel-self natural ligand [46]. In contrast with these reports, we observed diverse CDR3β sequences in Cr-induced ACD mice. This suggests that CDR3α rather than CDR3β is essential for recognition of Cr. We previously reported that Pd-specific TCR had high sequence identity with CDR3α but was diverse for CDR3β in Pd-induced ACD mice [17]. This similar result obtained with Pd-induced ACD mice suggests the relative significance of CDR3α to CDR3β is common to metal-induced allergy models in mice.

We observed shared CDR3α of VA11-1 among individual ACD mice, whereas identical CDR3β of VB14-1 was not observed among mice. This suggests CDR3α of VA11-1 and germline-encoded CDR1β and CDR2β of VB14-1 were used for interactions of Cr with TCR. It was reported that activation of Ni-specific SE9 T cells depended on Thr residues in CDR1α and CDR3α [47]. Also, Ni-specific ANi2.3 interactions depended on Tyr and Thr residues in CDR1α and a Phe residue in CDR2α, and that ANi2.3 has an Arg-Asp-Gly-Tyr (RDGY) motif in CDR3β [46]. In this study, we did not identify a motif for Cr-binding in CDR3α of VA11-1 but interestingly, shared TCR clonotypes contained Glu, Thr, His, Arg, and Tyr residues that have the potential to interact with Cr ions. Binding topology of metal-specific TCR to peptide-MHC is predicted to vary with metal species, MHC and self-antigen. Cr-specific TCR probably react with peptide-MHC complexes in a different manner than human Ni- or Be-specific TCRs.

We observed that the expression levels of Th1/Th2, TNF-α, TNF-R1, Fas, and Fas L were markedly induced in the footpad in response to Cr. IFN-γ and TNF-α produced by Th1 cells activate macrophages, and occasionally kill macrophages and other sensitive cells by Fas L signaling. Apoptosis of keratinocytes induced by T cells and mediated by IFN-γ and Fas is a crucial event in the transition from activation of the immune system to the manifestation of ACD [48]. Keratinocytes apoptosis describes activation-induced cell death, because IFN-γ up-regulates Fas and renders keratinocytes susceptible to apoptosis [49]. In addition, a previous study showed that TNF-α secreted by activated T cells contributed to spongiosis in ACD [50]. T cell-derived TNF-α is a sensitizer for Fas-mediated apoptosis [50]. TNF mediates its biological effects through TNF-R1 and TNF-R2 whereby TNF-induced apoptosis is largely mediated via TNF-R1. Our results suggest that apoptosis of keratinocytes induced by TNF-α is implicated in the pathogenesis of Cr allergy. The induction of keratinocytes apoptosis by skin-infiltrating T cells, subsequent cleavage of E-cadherin, and resisting desmosomal cadherins represent molecular events in spongiosis [51]. It is likely that Th1-type cytokines and cytotoxic molecules from NKT cells and Cr-specific T cells are positively correlated with the pathogenesis of metal allergy in mouse footpads.

In conclusion, we established a mouse model of Cr-induced ICD and ACD and found that allergen-specific T cells used a specific TCR repertoire in Cr-induced ACD. The infiltrated T cells included NKT cells and Cr-specific T cells with VA11-1/VB14-1 usage. This novel mouse model is useful for the study of the pathogenic roles of T cells in Cr allergy and the intriguing results obtained from this study will provide new insights into antigen specificity of TCRs and the role of TCRα chains in Cr-specific T cells. Further studies using this mouse model of metal allergy will contribute to the foundation for diagnosis of metal allergy and new treatments to control metal-specific T cells.

## Supporting Information

Table S1 **Alignment of amino acid sequences of CDR3a of VA11-1.** *: number of mice in which CDR3 sequence occurred. **: consensus amino acids are in bold. Glu (E), Thr (T), His (H), Arg (R), and Tyr (Y) are underlined.
(DOC)
