# Supplementary material for: Accumulation of Metal-Specific T Cells in Inflamed Skin in a Novel Murine Model of Chromium-Induced Allergic Contact Dermatitis
Source: PLoS One. 2014 Jan 20;9(1):e85983. doi: 10.1371/journal.pone.0085983 (PMC3896422; doi:10.1371/journal.pone.0085983)
Supplement: Table S1 — Alignment of amino acid sequences of CDR3a of VA11-1. *: number of mice in which CDR3 sequence occurred. **: consensus amino acids are in bold. Glu (E), Thr (T), His (H), Arg (R), and Tyr (Y) are underlined. (DOC) [file pone.0085983.s001.doc]

**Table S1. Alignment of amino acid sequences of CDR3a of VA11-1**

| Frequency* | VA | CDR3** | AJ |
| --- | --- | --- | --- |
| 4/5 | VA11-1 | **CAA** EAG**G**Y **K**V**VF** | AJ12 |
| 3/5 | VA11-1 | **CAA** GNT**G**NY**K**Y**VF** | AJ40 |
| 3/5 | VA11-1 | **CA**G HRG**S**ALGR**L**H**F** | AJ18 |
| 2/5 | VA11-1 | **CAA** TS**S**SFS**KLVF** | AJ50 |
| Consensus |  | **CAA**-XX**SG**XX**KLVF** |  |

*: number of mice in which CDR3 sequence occurred.

**: consensus amino acids are in bold. Glu (E), Thr (T), His (H), Arg (R), and Tyr (Y) are underlined.
